# Comparing flow and pulmonary artery growth post-patent ductus arteriosus stenting in patients with ductal-dependent pulmonary flow using 4D magnetic resonance imaging

**DOI:** 10.1093/ehjimp/qyae044

**Published:** 2024-05-14

**Authors:** Faiza A Al Kindi, Hamood Al Kindi, Madan Mohan Maddali, Abdullah Al Farqani, Khalid Al Alawi, Abdullah Al Balushi, Mohammed Al Ghafri, Sahar Khalil, Satish Kumar

**Affiliations:** The Radiology Department, The Royal Hospital, P.O. Box 1331, Ghala St, 111 Muscat, Oman; The National Heart Center, The Royal Hospital, P.O. Box 393, 18 November St, 100 Muscat, Oman; The National Heart Center, The Royal Hospital, P.O. Box 393, 18 November St, 100 Muscat, Oman; The National Heart Center, The Royal Hospital, P.O. Box 393, 18 November St, 100 Muscat, Oman; The National Heart Center, The Royal Hospital, P.O. Box 393, 18 November St, 100 Muscat, Oman; The National Heart Center, The Royal Hospital, P.O. Box 393, 18 November St, 100 Muscat, Oman; The National Heart Center, The Royal Hospital, P.O. Box 393, 18 November St, 100 Muscat, Oman; The Radiology Department, The Royal Hospital, P.O. Box 1331, Ghala St, 111 Muscat, Oman; Centre of Studies and Research, Ministry of Health, P.O. Box 393, 100 Muscat, Oman

**Keywords:** cardiac, cardiovascular system, MR angiography, decision analysis, diagnostic procedure, imaging sequences, congenital, 4D-flow MRI

## Abstract

**Aims:**

The 4D magnetic resonance imaging (4D-flow MRI) provides a qualitative and quantitative assessment of cardiovascular structures and processes. 4D-flow MRI was used to study pulmonary flow in post-patent ductus arteriosus (PDA) stent insertion in duct-dependent pulmonary flow neonates at baseline (PDA stent insertion) and after 6 months, and also, to evaluate the effect of flow dynamics on the growth of pulmonary arteries (PAs).

**Methods and results:**

This prospective observational study included neonates with ductus arteriosus-dependent pulmonary circulation who underwent ductal stenting between June 2021 and November 2022. Cardiac 4D-flow MRI and magnetic resonance angiography were conducted in two phases; after the deployment of the PDA stent during the neonatal period and after 6 months from stent deployment. Eight neonates were recruited, but only five completed both scans. A total of 10 PAs were evaluated during each phase. The median left PA (LPA) and right PA (RPA) diameters and indexed flow for LPA and RPA were evaluated. The growth rate of LPA was observed to be lower than that of RPA (percentage diameter increase: 74 vs. 153%). LPA *Z*-score was lower than RPA. Indexed flow in both LPA and RPA showed a reduction in the 6-month scan, which was consistent with reduced stent patency.

**Conclusion:**

4D-flow cardiac MRI showed different growth rates and reduced flow between LPA and RPA post-PDA stent. These insights can aid in future management decisions.

## Graphical Abstract

**Graphical Abstract:**
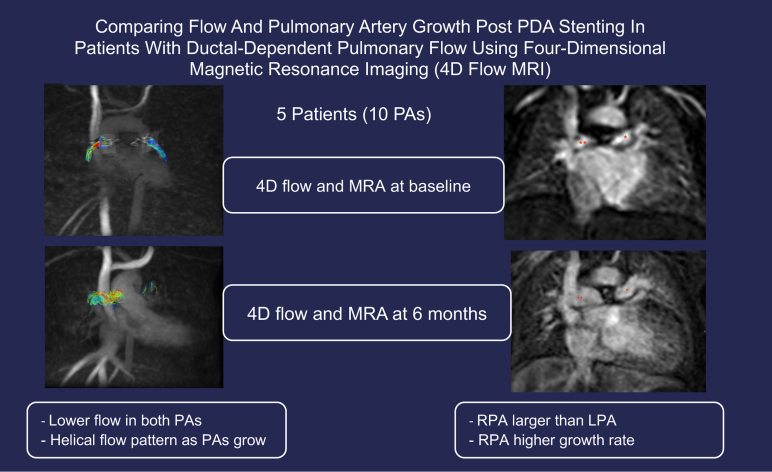

## Introduction

Cardiac abnormalities are among the most common forms of congenital disease, with a prevalence of 6–10 per 1000 births. Together, they represent a significant disease burden. Cardiac imaging provides an important adjunct to clinical evaluation, contributing to a precise diagnosis as well as providing an accurate means to monitor progression and guide treatment decisions in these children. Magnetic resonance imaging (MRI) is considered the gold standard imaging modality and has considerably improved structural and functional assessment and reduced the need for invasive procedures.^1–3^ The development of 4D-flow techniques offers the potential to further add to current capabilities. 4D flow provides a time-resolved 3D description of the total dynamics of blood flow, allowing comprehensive retrospective analysis of any anatomical location within the scanned heart chambers and vascular system.^4–7^

The paediatric population with complex congenital heart disease (CHD) perhaps represents one of the most important groups with the potential to benefit from the application of 4D flow. Many of the surgical interventions performed are associated with planned reoperations, such as staged repair of single-ventricle circulation and replacement of right ventricle-to-pulmonary artery (PA) conduits. Altered blood flow in this setting is postulated to contribute to deteriorating cardiac function. The quantitative nature of 4D-flow MRI is well placed to provide new insights into these processes on an individual as well as a disease-specific level.^8,9^

There has been a recent increase in interest in applying 4D flow to the paediatric congenital population. However, right heart and PA flow have been examined in a small number of individuals, both in the setting of tetralogy of Fallot and following the Ross procedure.^4,8^ The evaluation of complex dynamics in post-procedural shunt placements, such as patent ductus arteriosus (PDA) stents using MRI, has been shortly studied as well. The quantitative measurement of flow and how it relates to the overall development of cardiac structures is still an area to be explored. In our research, we aimed to study the pulmonary flow dynamics in the post-PDA stent procedure in patients with duct-dependent pulmonary flow using the novel 4D-flow MRI, and also, to evaluate the growth of PAs non-invasively and how flow and growth of PAs are related over a period of time.

## Methods

**Figure 1:**
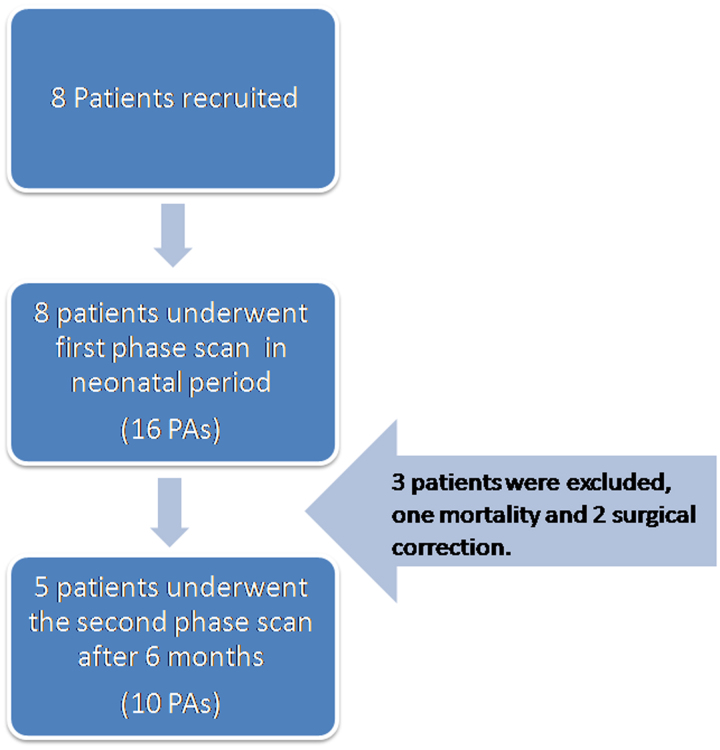
Summary of the patient recruitment.

This is a prospective observational study that included eight neonates with ductus arteriosus-dependent pulmonary circulation who underwent ductal stenting between June 2021 and November 2022. Neonates, who were haemodynamically unstable following the ductal stenting and those with additional antegrade pulmonary blood flow, were excluded from the study. *Figure 1* summarizes the patient selection.

All neonates received general anaesthesia following standard American Society of Anesthesia monitoring guidelines. Imaging was performed after stent deployment and around 6 months later.

Imaging was done using a 1.5 T MRI, Signa Artist (GE; GE Healthcare, Milwaukee, WI, USA). Post-processing was done by a Level 1 cardiac imaging radiologist and cardiologist. Images were acquired with retrospective ECG gating, 320 mm^3^ volume, 1.0–1.25 mm^3^ isotropic spatial resolution, and velocity encoding (VENC) of 150–200 cm/s. The post-processing was done using CMR42 CVI post-processing workstation (GE; GE Healthcare). Scanning parameters also included magnetic resonance angiography (MRA) sequences to assess the PAs in addition to the 4D-flow sequences. MRA and 4D-flow data were acquired after the bolus injection of 0.2–0.4 mmol/kg gadolinium-based contrast agent.

### Data analysis

The diameters, blood flow, and characteristics of the right PA (RPA) and left PA (LPA) were assessed. The comparison of means and data was treated to fit appropriately with the idiographic approach.

## Results

Eight neonates were recruited during the study period, and all of them had a baseline MRI study following the PDA stent insertion. There was one mortality, and two patients underwent a modified Blalock–Taussig shunt (MBTS) later during the follow-up period. Therefore, five patients had completed both the baseline and the 6-month follow-up scan. *Table 1* summarizes the baseline clinical features. The mean [standard deviation (SD)] birth weight was 3.03 kg (0.47). Seven out of eight patients had single-ventricle physiology. The PDA originated from the distal aortic arch in four patients and from the aortic isthmus in three patients. In only one patient, the PDA originated from the innominate artery. The mean (SD) oxygen saturation during the follow-up MRI scan was 70%.^9^ One patient underwent two ventricle repairs in the form of a Rastelli procedure, and five patients underwent Glenn shunt. Two patients underwent left pulmonary arterioplasty, and one patient required right pulmonary arterioplasty during the Glenn shunt procedure. One patient received MBTS following PDA stent insertion as a final palliative procedure.

**Table 1:** Demonstrating the baseline clinical features of the patients

Clinical variables	Mean (SD)/frequency
Birth weight (kg)	3.03 (0.47)
Gender	
Male	5
Female	3
Diagnosis	
CCTGA/PA	1
DORV/PA	1
MA/DORV/PA	1
PA IVS	2
PA/UBAVSD	2
PA/VSD	1
Aortic arch	
Left	6
Right	2
Single-ventricle pathway	
No	1
Yes	7
O_2_ saturation % on discharge	88 (5)
Origin of the PDA	
Distal aortic arch	4
Innominate artery	1
Isthmus	3
Number of the PDA stent	
1	5
2	3
Other procedures during first PDA stent	
Atrial septostomy	3
None	5
Subsequent PDA stent Cath reintervention	
No	5
Yes	1
O_2_ saturation % on the follow-up MRI scan	70 (9)
Cardiac surgical procedures	
MBTS following PDA stent	2
Glenn shunt	5
Right pulmonary arterioplasty	1
Left pulmonary arterioplasty	2
Rastelli procedure	1
Outcome	
Died	1
Alive	7

CTGA/PA, congenitally corrected transposition of the great arteries/pulmonary atresia; DORV/PA, double outlet right ventricle/pulmonary atresia; MA/DORV/PA, mitral atresia/double outlet right ventricle/pulmonary atresia; PA/IVS, pulmonary atresia/intact ventricular septum; PA/UBAVSD, pulmonary atresia/unbalanced atrioventricular septal defect; PA/VSD, pulmonary atresia/ventricular septal defect.

### Qualitative 4D-flow MRI analysis

**Figure 2:**
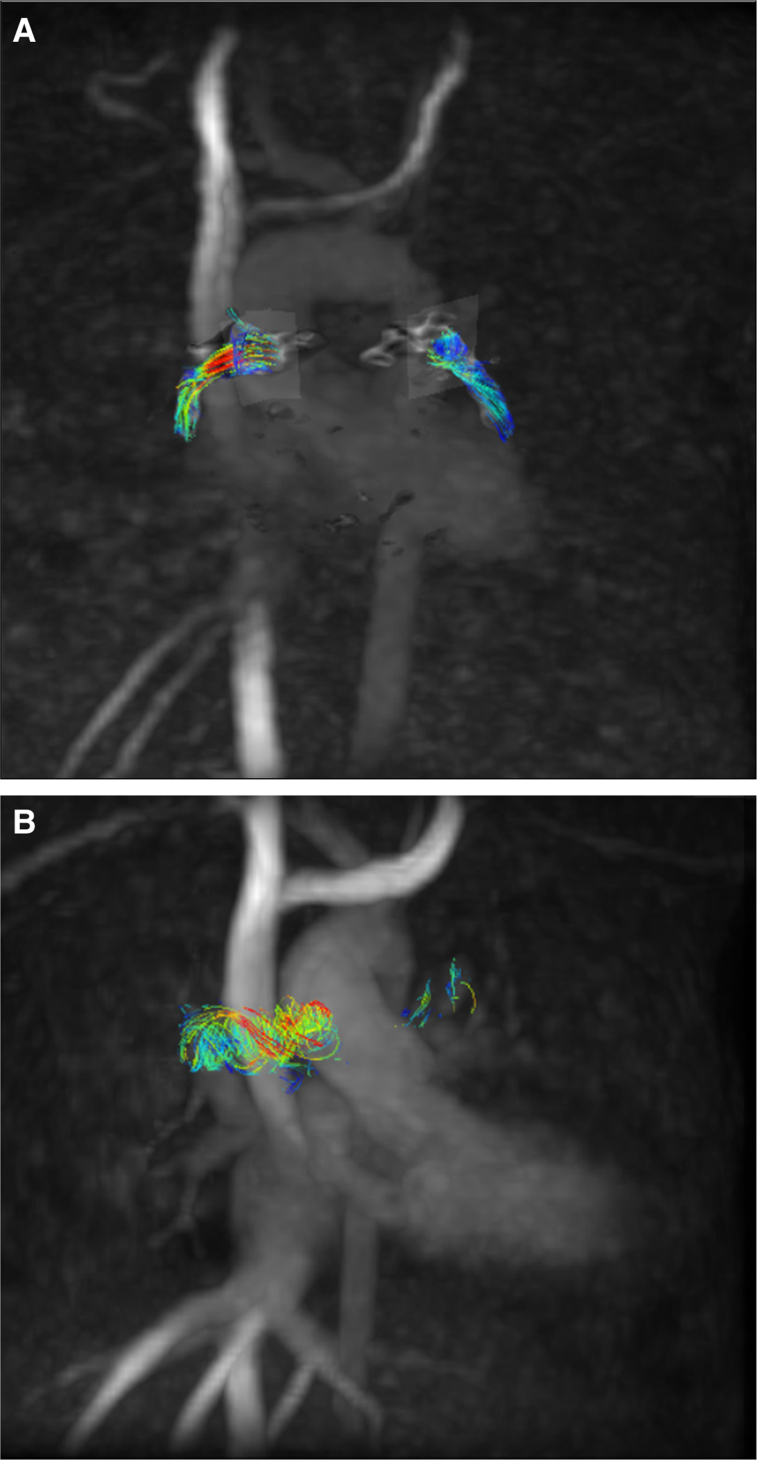
The 4D-flow image of flow streamlines in LPA and RPA, at baseline (*A*) and after 6 months from deploying the stent (*B*), demonstrating the change in flow pattern from linear to helical and the density of streamlines owed by the reduction in flow after 6 months.

*Figure 2A* and *B* illustrate the streamline flow pattern in the right and LPAs after PDA stent insertion during the immediate and 6-month follow-up period. The flow pattern in the first scan was linear, but it has changed to a helical pattern in the 6-month follow-up, particularly noted in the larger RPA. However, the streamlines were fewer in the 6 months denoting less flow.

### Quantitative 4D-flow MRI analysis

The mean heart rate, weight, height, and body surface area of the patients in the first and second MRI scans are summarized in *Tables 2*. *Table 3* summurizes the parameters for each of the LPA and RPA obtained from the 4D flow quantification, diameters obtained from the MRA and also the parameters obtained by echocardiography done at same time. *Table 4* provides a comparison of LPA and RPA parametes at exh scan phase.

**Table 2:** Summury of patients parameters

	Baseline (*n* = 8)	After 6 months (*n* = 5)	Test	df	*P*-value	95% CI	Interpretation
Weight (kg)	3.15 ± 0.47	6.42 ± 1.33	6.478	11	0.0001	(−4.3809 to −2.1591)	*P* < 0.001
Height (cm)	50.625 ± 2.19	65.60 ± 2.51	11.364	11	0.0001	(−17.87536 to −12.07464)	*P* < 0.001
BSA	0.2 ± 0	0.322 ± 0.49	0.724	11	0.484	(−0.49276 to 0.24876)	NS
Heart rate (BPM)	143.75 ± 10.60	126 ± 23.02	1.91	11	0.0818	(−2.6451 to 38.1451)	NS

**Table 3:** LPA and RPA parameters measured from MRI and echocardiography

	Mean ± SD Baseline (*n* = 8)	Mean ± SD After 6 months (*n* = 5)	*T*-test	df	*P*-value	95% CI	Interpretation
Left pulmonary artery							
Diameter (mm)	3.56 ± 1.4	6.84 ± 1.5	4.003	11	0.0021	(−5.0833 to −1.4767)	*P* < 0.001
Flow (mL/cycle)	1.15 ± 0.6	1.02 ± 0.4	0.4255	11	0.678	(−0.5425 to 0.8025)	NS
Flow indexed	0.82 ± 0.4	0.39 ± 0.1	2.322	11	0.0404	(0.0225 to 0.8375)	*P* < 0.05
Max *V* (cm/s)	79.48 ± 37.8	56.66 ± 49.4	0.944	11	0.3653	(−30.3654 to 76.0054)	NS
WSS (Pa)	0.39 ± 0.3	0.2 ± 0.1	1.35	11	0.204	(−0.1197 to 0.4997)	NS
Diameter by ECHO	3.66 ± 0.7	6.4 ± 1.1	5.543	11	0.0002	(−3.8280 to −1.6520)	*P* < 0.001
*Z-*score by ECHO	−1.47 ± 0.8	−0.08 ± 1	2.777	11	0.018	(0.2883 to 2.4917)	*P* < 0.05
Right pulmonary artery							
Diameter (mm)	3.82 ± 0.7	9.16 ± 2.4	6.0384	11	0.0001	(−7.2864 to −3.3936)	*P* < 0.001
Flow (mL/cycle)	2.42 ± 1.2	2.35 ± 0.6	0.12	11	0.9067	(−1.2141 to 1.3541)	NS
*F* index	1.73 ± 0.9	0.96 ± 0.3	1.824	11	0.0954	(−0.1590 to 1.6990)	NS
WSS (Pa)	0.58 ± 0.3	0.52 ± 0.5	0.273	11	0.7896	(−0.4230 to 0.5430)	NS
RPA diameter by ECHO	3.82 ± 0.7	8.58 ± 2.7	4.85	11	0.0005	(−6.9198 to −2.6002)	*P* < 0.001
RPA *Z-*score by ECHO	−1.49 ± 0.6	1.78 ± 2.5	6.326	11	0.004	(−5.2547 to −1.2853)	*P* < 0.05

**Table 4:** Comparison of LPA and RPA parametes at each scan phase

	Mean ± SD LPA	Mean ± SD RPA	*T-*test	df	*P* value	CI	Interpretation
first scan (*n* = 8)							
Diameter (mm)	3.56 ± 1.4	3.82 ± 0.7	0.4698	14	0.654	(−1.4469 to 0.9269)	NS
Flow (mL/cycle)	1.15 ± 0.6	2.42 ± 1.2	2.6774	14	0.018	(−2.2874 to −0.2526)	*P* < 0.05
*F* indexed	0.82 ± 0.4	1.73 ± 0.9	2.6134	14	0.0204	(−1.6568 to −0.1632)	*P* < 0.05
WSS (Pa)	0.39 ± 0.3	0.58 ± 0.3	1.2667	14	0.2259	(−0.5117 to 0.1317)	NS
Diameter by ECHO	3.66 ± 0.7	3.82 ± 0.7	0.4571	14	0.6546	(−0.9107 to 0.5907)	NS
*Z*-score by ECHO	−1.47 ± 0.8	−1.49 ± 0.6	0.0566	14	0.9557	(−0.7383 to 0.7783)	NS
6 months follow up scan (*n* = 5)							
Diameter (mm)	6.84 ± 1.5	9.16 ± 2.4	1.833	8	0.1042	(−5.2387 to 0.5987)	NS
Flow (mL/cycle)	1.02 ± 0.4	2.35 ± 0.6	4.1242	8	0.0033	(−2.0737 to −0.5863)	*P* < 0.01
*F* indexed	0.39 ± 0.1	0.96 ± 0.3	4.0305	8	0.0038	(−0.8961 to −0.2439)	*P* < 0.01
WSS (Pa)	0.2 ± 0.1	0.52 ± 0.5	1.4033	8	0.1981	(−0.8459 to 0.2059)	NS
Diameter by ECHO	6.4 ± 1.1	8.58 ± 2.7	1.672	8	0.1331	(−5.1867 to 0.8267)	NS
*Z*-score by ECHO	−0.08 ± 1	1.78 ± 2.5	1.5446	8	0.161	(−4.638 to 0.9168)	NS

The LPA diameter in the first scan was 3.56 ± 1.4 mm and increased significantly in the follow-up MRI scan to 6.84 ± 1.5 mm (*P* < 0.001). The indexed flow in the LPA reduced significantly between the two scans from 0.82 ± 0.4 to 0.39 ± 0.1 (*P* < 0.05). The maximum velocity and the wall shear stress (WSS) of the LPA were reduced between the two scans, but they were not statistically significant.

The RPA diameter in the first scan was 3.82 ± 0.7 mm and increased significantly in the follow-up MRI scan to 9.16 ± 2.4 mm (*P* < 0.001). The indexed flow in the RPA changed between the two scans (1.73 ± 0.9–0.96 ± 0.3, *P* > 0.05). In addition, the maximum velocity and the WSS of the RPA did not change between the two scans.

### Comparison between the LPA and RPA

**Figure 3:**
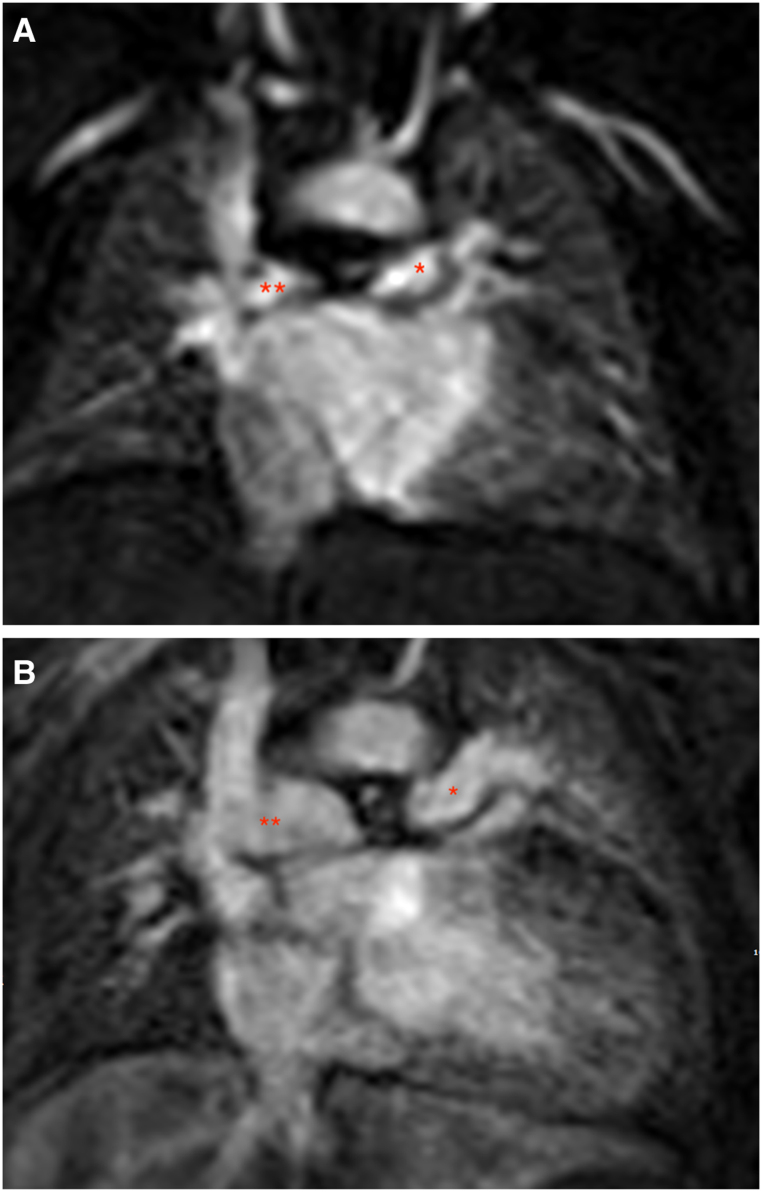
The MRA image of LPA and RPA, at baseline (*A*) and after 6 months of deploying the stent (*B*), demonstrating the change diameter. Notice the larger RPA in the images at 6 months (*B*). The 2 asterisks are marking the RPA and one asterisk is making the LPA.

**Figure 4:**
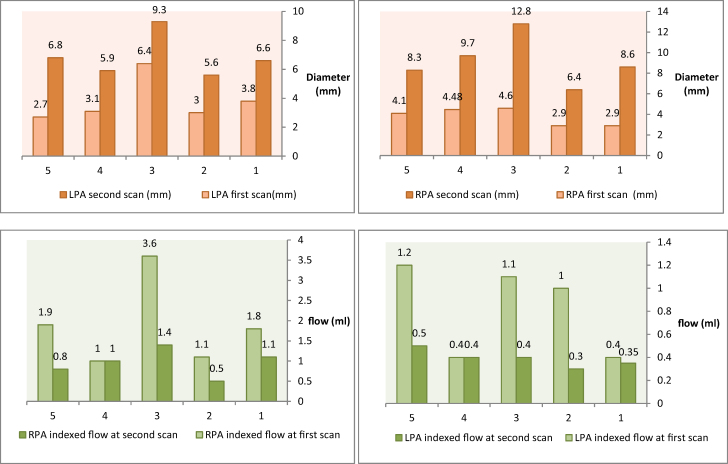
Graphs demonstrating the (*A*) growth of LPA and RPA for the five patients labelled from 1 to 5, at both the first scan (neonatal period) and after 6 months, measured by mm (Top 2 bar graphs) and (*B*) change of the flow measured from 4D-flow MRI, of LPA and RPA for the five patients labelled from 1 to 5, at both the first scan (neonatal period) and after 6 months, measured by mL (Lower 2 bar graphs).

Six months following the PDA stent insertion, the RPA diameter was noted to be larger than the LPA diameter (9.16 ± 2.4 vs. 6.84 ± 1.5 mm) but it was not statistically significant (*P* > 0.05). The maximum velocity and the WSS were similar between the LPA and RPA in both scans (*Table 4*). However, the flow and the indexed flow were significantly lower in the LPA compared with the RPA in both scans. *Figure 3A* and *B* demonstrates the change in diameter from baseline. *Figure 4* demonstrates the growth and flow differences of the LPA and RPA between the two scans.

## Discussion

Our study identified distinct growth patterns in the RPA and LPA following PDA stenting. We found that the RPA had a higher growth rate compared with the LPA. The original hypothesis that both PAs would grow similarly after PDA stent deployment was rejected, and an alternate hypothesis was accepted. These findings are consistent with the findings of Santoro *et al*. who studied post-PDA stenting growth patterns in patients with pulmonary-dependent circulation. Santoro *et al.* used interventional procedures to calculate diameters. Our study suggests that MRA could be a non-invasive alternative to interventional studies for monitoring the growth and development of PAs in these patients.

The growth and flow dynamics of PAs in children with CHD are of paramount clinical importance, influencing both diagnosis and management. Recent studies have explored the potential of 4D-flow MRI for evaluating flow dynamics in various cardiac structures. Our study focused on using the 4D-flow MRI and MRA in the quantitative assessment of flow and growth in the LPA and RPA at baseline, at the time of PDA stent placement, and at 6-month follow-up. There have been recent studies looking into the applications of this modality in CHD. Valverde *et al*.^10^ utilized 4D-flow MRI to quantify flow in systemic-to-pulmonary collaterals, showcasing the technique’s applicability. Markl *et al*.^5,8^ investigated haemodynamic changes in children with single-ventricle physiology, emphasizing the significance of understanding PA growth patterns in optimizing surgical strategies for these patients.

Our study revealed distinctive growth patterns in the RPA and LPA following PDA stenting. The RPA exhibited a more substantial growth rate compared with the LPA, which aligns with findings by Santoro *et al*.^11,12^ who studied post-PDA stenting growth patterns in patients with pulmonary-dependent circulation. In their study, interventional procedures were used as the modality to calculate diameters. Our study suggests that MRA could serve as a non-invasive alternative to interventional studies for monitoring the growth and development of PAs in these patients.

The deployment of PDA stents has been proven to promote and improve circulation and growth of PAs in many studies.^13,14^ This has been postulated to be a factor for PA growth and development in virtue of increased flow. A multicentre comparison study of palliative PDA stent and MBTS showed that PAs were larger and measured by the Nakata index and more symmetrical in the PDA stent group.^15^ However, the absolute quantitative measurements of flow in the PAs have not been fully studied. In our study, we found a global decrease in the quantity of flow in all 10 PAs studied for the five recruited patients in the 6-month follow-up compared with the baseline. Sivakumar^16^ found a gradual decrease in oxygen levels caused by the growth of tissue within the ductal stents in their study of neonatal PDA stents. They also demonstrated that the cause was due to the development of a layer of fibrous tissue and blood clot within surgical shunts. The latter finding could explain the global quantitative reduction in the flow in all PAs in our study, and it may also concur with the previous postulations about the longevity of PDA stents in this population. The papers provide insights into the longevity of PDA stents. Alwi *et al.*^14^ found that PDA stenting was successful in a majority of patients with duct-dependent pulmonary circulation, but there were cases of stent stenosis and worsening branch PA stenosis during follow-up. Rodríguez-Cruz *et al*.^17^ presented two cases where stents were placed in the PDA, but did not provide specific information on long-term patency. Wespi *et al*.^18^ reported favourable short- to mid-term outcomes after PDA stenting, with successful procedural outcomes and adequate vessel growth for subsequent surgical procedures. However, the study did not include a long-term follow-up. Overall, it is suggested that PDA stenting can be a feasible and safe treatment option, but long-term patency and potential complications need further investigation.

The discrepancy in diameter growth, despite reductions in flow in both arteries, raises questions about factors beyond quantitative flow, such as vortices and flow patterns, playing a role in PA growth. Evidence from studies on bicuspid aortic and pulmonary valve patients supports the idea that flow patterns play a crucial role in vessel dilatation.^19–22^ Our study noted helical streamlines, particularly in the RPA at the 6-month follow-up, a phenomenon also found in normal RPA by Bächler *et al*.^23^ Gbinigie *et al.*^24^ described flow asymmetry in the PAs found in healthy individuals. A right-handed helical pattern is observed in the main PA. Beyond the bifurcation, helical flow with positive helicity is found in the RPA and with negative helicity in the LPA. It was also found that the RPA is 10% larger than the LPA in diameter. Moreover, the average cardiac cycle flow velocity was 40% larger in the RPA compared with the LPA.

The observed increase in LPA diameter, associated with a more significant reduction in indexed flow, suggests that PDA stenting may exert a more pronounced effect on the left pulmonary circulation. This information has critical implications for post-intervention management. It aligns with previous observations of LPA augmentation during surgical intervention for patients with ductal stenting, reinforcing the idea that PDA stenting significantly influences lower flow dynamics in the LPA.^25^

The limitations of the study include its small sample size and the need for further, larger research to validate the findings. Also, the study would benefit from longer term follow-up to assess the progression of the observed changes. One other limitation is the requirement of intensive care admission and the need for anaesthesia and intubation to perform the MRI. The recruitment of these resources was a challenge due to the occupancy issue. The small size of the PAs in these neonates was a challenge to image. It required a very small matrix and high resolution with a small slice thickness which rendered the scans very large in size and, therefore, more challenging for analysis of the 4D flow. A last limitation is the absence of reference of normal quantitative flow of PAs calculated from MRI. Therefore, we used the echocardiography *Z*-score as a reference.

## Conclusion

The utilization of 4D-flow CMR in assessing the impact of PDA stenting on neonates with ductal-dependent pulmonary flow provides valuable insights into the haemodynamic changes and structural adaptations following this intervention. The study’s findings highlight the importance of MRI techniques, in particular, 4D-flow technique, in improving our understanding of CHDs and guiding clinical decision-making.

## Consent

Informed consent was obtained from the parents of all recruited patients, and institutional ethical committee approval was also obtained.

## Data Availability

The data underlying this article will be shared on reasonable request with the corresponding author.

## Lead author biography

Faiza A Al Kindi, MD, FRCPS(C), Diplomat ABR Sr Consultant Cardiac Radiologist Senior consultant in cardiac and thoracic imaging at the Royal hospital since 2010. Program director of the radiology residency program under the auspicious of Oman Medical Board of Specialty from 2014 till 2021. Assigned The assistant director of the National Heart center at the Royal Hospital from 2017 till 2022.

## References

[1] Beerbaum P, Körperich H, Barth P, Esdorn H, Gieseke J, Meyer H. Noninvasive quantification of left-to-right shunt in pediatric patients: phase-contrast cine magnetic resonance imaging compared with invasive oximetry. Circulation 2001;103:2476–82. 11369688 10.1161/01.cir.103.20.2476

[2] Debl K, Djavidani B, Buchner S, Heinicke N, Poschenrieder F, Feuerbach S et al Quantification of left-to-right shunting in adult congenital heart disease: phase-contrast cine MRI compared with invasive oximetry. Br J Radiol 2009;82:386–91. 19153187 10.1259/bjr/18500608

[3] Hsiao A, Lustig M, Alley MT, Murphy M, Chan FP, Herfkens RJ et al Rapid pediatric cardiac assessment of flow and ventricular volume with compressed sensing parallel imaging volumetric cine phase-contrast MRI. AJR Am J Roentgenol 2012;198:W250–9. 22358022 10.2214/AJR.11.6969 PMC3515670

[4] Geiger J, Markl M, Jung B, Grohmann J, Stiller B, Langer M et al 4D-MR flow analysis in patients after repair for tetralogy of Fallot. Eur Radiol 2011;21:1651–7. 21720942 10.1007/s00330-011-2108-4

[5] Markl M, Frydrychowicz A, Kozerke S, Hope M, Wieben O. 4D flow MRI. J Magn Reson Imaging 2012;36:1015–36. 23090914 10.1002/jmri.23632

[6] Vasanawala SS, Hanneman K, Alley MT, Hsiao A. Congenital heart disease assessment with 4D flow MRI. J Magn Reson Imaging 2015;42:870–86. 25708923 10.1002/jmri.24856

[7] Markl M, Schnell S, Wu C, Bollache E, Jarvis K, Barker AJ et al Advanced flow MRI: emerging techniques and applications. Clin Radiol 2016;71:779–95. 26944696 10.1016/j.crad.2016.01.011 PMC4930408

[8] Markl M, Geiger J, Jung B, Hirtler D, Arnold R. Noninvasive evaluation of 3D hemodynamics in a complex case of single ventricle physiology. J Magn Reson Imaging 2012;35:933–7. 22271353 10.1002/jmri.22861

[9] Lawley CM, Broadhouse KM, Callaghan FM, Winlaw DS, Figtree GA, Grieve SM. 4D flow magnetic resonance imaging: role in pediatric congenital heart disease. Asian Cardiovasc Thorac Ann 2018;26:28–37. 28185475 10.1177/0218492317694248

[10] Valverde I, Nordmeyer S, Uribe S, Greil G, Berger F, Kuehne T et al Systemic-to-pulmonary collateral flow in patients with palliated univentricular heart physiology: measurement using cardiovascular magnetic resonance 4D velocity acquisition. J Cardiovasc Magn Reson 2012;14:25. 22541134 10.1186/1532-429X-14-25 PMC3438058

[11] Santoro G, Capozzi G, Capogrosso C, Mahmoud HT, Gaio G, Palladino MT et al Pulmonary artery growth after arterial duct stenting in completely duct-dependent pulmonary circulation. Heart 2016;102:459–64. 26830664 10.1136/heartjnl-2015-308493

[12] Santoro G, Gaio G, Palladino MT, Iacono C, Carrozza M, Esposito R et al Stenting of the arterial duct in newborns with duct-dependent pulmonary circulation. Heart 2008;94:925–9. 17664187 10.1136/hrt.2007.123000

[13] Elmarsafawy H, Elasfar A, Taha FA. Evaluation of the growth of central pulmonary arteries following patent ductus arteriosus stenting in patients with duct dependent pulmonary circulation. Pediatr Cardiol 2020;41:1667–74. 32720086 10.1007/s00246-020-02426-8

[14] Alwi M, Choo KK, Latiff HA, Kandavello G, Samion H, Mulyadi MD. Initial results and medium-term follow-up of stent implantation of patent ductus arteriosus in duct-dependent pulmonary circulation. J Am Coll Cardiol 2004;44:438–45. 15261945 10.1016/j.jacc.2004.03.066

[15] Glatz AC, Petit CJ, Goldstein BH, Kelleman MS, McCracken CE, McDonnell A et al Comparison between patent ductus arteriosus stent and modified Blalock–Taussig shunt as palliation for infants with ductal-dependent pulmonary blood flow: insights from the congenital catheterization research collaborative. Circulation 2018;137:589–601. 29042354 10.1161/CIRCULATIONAHA.117.029987

[16] Sivakumar KB. PDA stenting in duct-dependent pulmonary circulation. In: Butera G, Chessa M, Eicken A, Thomson J, eds. Cardiac Catheterization for Congenital Heart Disease. Springer Link; 2019. p139–144.

[17] Rodríguez-Cruz E, Rosario-Pagán G, Muñoz-Rosario A, Duque-Salorziano S. Long term patency of the ductus arteriosus after stent placement: report of two cases and review of the literature. Bol Asoc Med P R 2007;99:40–3. 17616045

[18] Wespi R, Callegari A, Quandt D, Logoteta J, von Rhein M, Kretschmar O et al Favourable short- to mid-term outcome after PDA-stenting in duct-dependent pulmonary circulation. Int J Environ Res Public Health 2022;19:12794. 36232092 10.3390/ijerph191912794 PMC9566406

[19] Meierhofer C, Schneider EP, Lyko C, Hutter A, Martinoff S, Markl M et al Wall shear stress and flow patterns in the ascending aorta in patients with bicuspid aortic valves differ significantly from tricuspid aortic valves: a prospective study. Eur Heart J Cardiovasc Imaging 2013;14:797–804. 23230276 10.1093/ehjci/jes273

[20] Bissell MM, Loudon M, Neubauer S, Myerson SG. Abnormal haemodynamic flow patterns in bicuspid. Front Physiol 2017;8:374. 28620320 10.3389/fphys.2017.00374 PMC5449663

[21] Guzzardi DG, Barker AJ, van Ooij P, Malaisrie SC, Puthumana JJ, Belke DD et al Valve-related hemodynamics mediate human bicuspid aortopathy: insights from wall shear stress mapping. J Am Coll Cardiol 2015;66:892–900. 26293758 10.1016/j.jacc.2015.06.1310 PMC4545965

[22] Jayendiran R, Campisi S, Viallon M, Croisille P, Avril S. Hemodynamics alteration in patient-specific dilated ascending thoracic aortas with tricuspid and bicuspid aortic valves. J Biomech 2020;110:109954. 32827782 10.1016/j.jbiomech.2020.109954

[23] Bächler P, Pinochet N, Sotelo J, Crelier G, Irarrazaval P, Tejos C et al Assessment of normal flow patterns in the pulmonary circulation by using 4D magnetic resonance velocity mapping. Magn Reson Imaging 2013;31:178–88. 22898700 10.1016/j.mri.2012.06.036

[24] Gbinigie H, Coats L, Parikh JD, Hollingsworth KG, Gan L. A 4D flow cardiovascular magnetic resonance study of flow asymmetry and haemodynamic quantity correlations in the pulmonary artery. Physiol Meas 2021;42:025005. 33482652 10.1088/1361-6579/abdf3b

[25] Haranal M, Mood MC, Leong MC, Febrianti Z, Abdul Latiff H, Samion H et al Impact of ductal stenting on pulmonary artery reconstruction in patients with duct-dependent congenital heart diseases-an institutional experience. Interact Cardiovasc Thorac Surg 2020;31:221–7. 32437520 10.1093/icvts/ivaa069

